# A Guide to Structureless Visual Localization

**DOI:** 10.1007/s11263-026-02780-9

**Published:** 2026-05-09

**Authors:** Vojtech Panek, Qunjie Zhou, Yaqing Ding, Sérgio Agostinho, Zuzana Kukelova, Torsten Sattler, Laura Leal-Taixé

**Affiliations:** 1 Czech Institute of Informatics, Robotics and Cybernetics, Czech Technical University in Prague, Prague, Czech Republic; 2 Faculty of Electrical Engineering, Czech Technical University in Prague, Prague, Czech Republic; 3 NVIDIA, Santa Clara, California USA; 4 Visual Recognition Group, Faculty of Electrical Engineering, Czech Technical University in Prague, Prague, Czech Republic

**Keywords:** Visual Localization, Pose Regression, Pose Triangulation

## Abstract

Visual localization algorithms, i.e., methods that estimate the camera pose of a query image in a known scene, are core components of many applications, including self-driving cars and augmented / mixed reality systems. State-of-the-art visual localization algorithms are structure-based, i.e., they store a 3D model of the scene and use 2D-3D correspondences between the query image and 3D points in the model for camera pose estimation. While such approaches are highly accurate, they are also rather inflexible when it comes to adjusting the underlying 3D model after changes in the scene. Structureless localization approaches represent the scene as a database of images with known poses and thus offer a much more flexible representation that can be easily updated by adding or removing images. Although there is a large amount of literature on structure-based approaches, there is significantly less work on structureless methods. Hence, this paper is dedicated to providing the, to the best of our knowledge, first comprehensive discussion and comparison of structureless methods. Extensive experiments show that approaches that use a higher degree of classical geometric reasoning generally achieve higher pose accuracy. In particular, approaches based on classical absolute or semi-generalized relative pose estimation outperform very recent methods based on pose regression by a wide margin. Compared with state-of-the-art structure-based approaches, the flexibility of structureless methods comes at the cost of (slightly) lower pose accuracy, indicating an interesting direction for future work.

## Introduction

Visual localization is the task of predicting the position and orientation, *i.e.*, the camera pose, from which a given image was taken. Solving the visual localization task is a core step in many applications, including autonomous robots such as self-driving cars and drones, and augmented / mixed reality applications (Heng et al., 2018; Lim et al., 2012; Lynen et al., 2015; Middelberg et al., 2014; Panek et al., 2022).

State-of-the-art visual localization algorithms use 2D-3D matches between pixels in a query image and 3D points in the scene to estimate the camera pose (Panek et al., 2022; Sarlin et al., 2019; Sattler et al., 2017; Taira et al., 2021; Wang et al., 2024; Zhou et al., 2022). These matches are typically either established by matching local feature descriptors (Li et al., 2012; Panek et al., 2022, 2023; Sarlin et al., 2019, 2020; Sattler et al., 2017) or regressed directly via a neural network (Brachmann et al., 2023; Brachmann & Rother, 2020; Cavallari et al., 2019; Shotton et al., 2013). Given such 2D-3D matches, the camera pose can then be estimated by applying a perspective-n-point (PnP) pose solver (Grunert, 1841), *e.g.*, a P3P solver for calibrated cameras (Ding et al., 2023; Fischler & Bolles, 1981; Haralick et al., 1994; Persson & Nordberg, 2018), inside a RANSAC framework (Baráth et al., 2019; Chum & Matas, 2008; Fischler & Bolles, 1981; Lebeda et al., 2012). Alternatively, the camera pose can also be estimated by refining an initial pose estimate, *e.g.*, obtained from image retrival (Jégouetal., 2010; Arandjelovic & Zisserman, 2013); Arandjelović et al., 2015; (Berton et al., 2023; Gordo et al., 2017; Revaud et al., 2019), via a render-and-compare approach (Botashev et al., 2024; Chen et al., 2023, 2022; Lin et al., 2020; Liu et al., 2024, 2023; Pietrantoni et al., 2024, 2023; Sarlin et al., 2021; Sun et al., 2023; Trivigno et al., 2024; Zeller, 2024; Zhou et al., 2024): Given a current pose estimate, the query image is compared to a rendering of the scene (either in the form of colors or by rendering a feature space). The pose is then optimized as to increase the consistency between the actual image and the rendering.

Common to these approaches is that they represent scenes via 3D models, either storing the models explicitly, *e.g.*, in the form of Structure-from-Motion (SfM) point clouds, meshes, or 3D Gaussian Splats (Kerbl et al., 2023), or implicitly, *e.g.*, in the weights of a neural network or a neural radiance field (NeRF) (Mildenhall et al., 2020). While 3D model-based scene representations facilitate highly accurate camera pose estimation, they are also rather inflexible. In the case of changes in the scene, *e.g.*, a newly constructed building, a building being renovated, furniture being moved or replaced, *etc.*, detecting the changes (Adam et al., 2022; Yew & Lee, 2021) and updating the 3D model accordingly are typically complex tasks by themselves (Torii et al., 2021). Even simple operations such as switching to a different type of local features can lead to hours of downtime, where a structure-based localization system becomes unavailable as its underlying scene representation is being updated.

An alternative to the structure-based approaches discussed above are structureless visual localization methods (Arnold et al., 2022; Balntas et al., 2018; Bhayani et al., 2021; Dong et al., 2023; Laskar et al., 2017; Zhang & Kosecka, 2006; Zhou et al., 2019). Structureless approaches represent the scene through a database of images, each associated with a camera pose and camera intrinsics.^1^ Given a query image, they first use image retrieval to identify a set of relevant database images. They then estimate the camera pose of the query relative to the poses of the top retrieved images (Arnold et al., 2022; Bhayani et al., 2021; Kazhdan & Hoppe, 2013; Zheng & Wu, 2015; Zhou et al., 2019). Since each database image is treated independently of the other, adding images with new observations or deleting images that show outdated versions of a scene is trivial.^2^ Storing posed images is also usually less memory intensive than storing a 3D model.

Structureless methods are advantageous when we can obtain accurate reference image poses without running SfM. One of such situations might arise when using a hardware platform with precise localization, *e.g.*, through RTK (Real-time kinematic positioning), to capture the reference images. Another use case is the gradual extension of existing maps (Panek et al., 2025), where the structure-based method is used for localization in areas with enough 3D information, and the structureless method is used on the borders of the mapped area or in newly mapped areas, without a reconstructed 3D structure. The two approaches can even be combined into a single hybrid approach (Panek et al., 2025). This application is also supported by the observation that visual localization using structureless methods is more robust in the areas sparsely covered by reference images. Compared to structure-based methods, structureless methods are much faster to deploy in practice, as there is no need to reconstruct the 3D scene in the first place. This can be important in scenarios where rapid deployment is highly important. For example, in disaster scenarios, robots can first explore a place to determine which areas are safe and where human first-responders are most urgently needed. The human teams can then use this map to localize themselves and thus navigate. The same speed advantage holds in cases when the scene representation has to be updated, *e.g.*, buildings torn down or built, furniture moved or replaced. The whole update consists of removing images capturing the affected part of the scene and adding new ones. Changing to a different type of local feature or matcher is also easier, as there is no 3D structure in the scene representation, which would need to be recomputed.

Structureless methods were among the first visual localization approaches (Zhang & Kosecka, 2006). However, there is significantly less work on structureless methods than on structure-based methods. Interestingly, a large part of the literature on structure-based methods does not compare with structureless approaches. At the same time, to the best of our knowledge, there is not even a comprehensive comparison of structureless methods between themselves. This paper aims to close this gap in the literature.

**Fig. 1:**
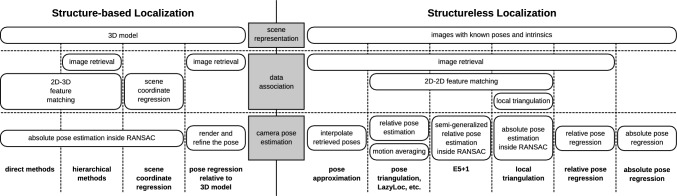
Comparison chart for structure-based and structureless methods described in Sect. 2

In detail, this paper makes the following contribution: (**1**) we provide a comprehensive review of structureless localization methods. (**2**) through extensive experiments, we compare state-of-the-art versions of the most important families of structureless methods: pose triangulation, semi-generalized relative pose estimation, absolute pose estimation via locally triangulated 3D point clouds, and relative pose regression. For each method, we first ablate multiple variants (mostly by evaluating the use of different local features). We then compare between the different families using the best-performing version from the previous experiments. (**3**) the results of our experiments lead to multiple interesting insights: (*a*) More explicit use of geometric reasoning often leads to better pose accuracy. *I.e.*, methods relying on pose triangulation from relative poses perform worse than methods based on semi-generalized relative pose estimation (which estimates the pose of the query simultaneously *w.r.t.* multiple database images instead of computing only pairwise relative poses). In turn, methods based on locally triangulating the 3D scene structure followed by absolute pose estimation can provide more accurate pose predictions than approaches based on semi-generalized relative pose estimation. Interestingly, relative pose regression-based approaches perform worst, despite strong recent progress (Dong et al., 2024; Leroy et al., 2024; Wang et al., 2024). (*c*) In terms of their pose accuracy-run-time trade-off, methods using less geometric reasoning can offer a better performance, *i.e.*, methods that do not provide the highest pose accuracy are still useful in practice. (*d*) There is no type of features that performs best in all scenarios. The best type of feature to use depends on the method and dataset. (**4**) We provide a comparison of the best-performing structureless methods with state-of-the-art structure-based approaches. Our results show that structureless approaches can be competitive with structure-based methods. Thus, improving the accuracy of structureless localization algorithms is an interesting direction for future work.

## Related Work

As this paper focuses on comparing structureless approaches for visual localization, we focus our discussion on such methods. For completeness, we also review structure-based approaches. We present a comparison chart for selected structure-based and structureless method in Fig. 1.

### Place Recognition

Highly related to the (structureless) visual localization problem is the task of visual place recognition (Baatz et al., 2010, 2011; Berton et al., 2022, 2023; Chen et al., 2011, 2017; Hausler et al., 2021; Lowry et al., 2016; Torii et al., 2015); Arandjelović & Zisserman 2014; Singh & Košecká 2016; (Ardeshir et al., 2014; Cao & Snavely, 2013; Zamir & Shah, 2010, 2014). Given a query image and a database of geo-tagged photos, the goal of place recognition is to identify the scene depicted in the query. This is typically done by retrieving a database image depicting the same place. Place recognition approaches thus build on image retrieval techniques Arandjelović et al., 2015;(Berton et al., 2022, 2023; Radenovic et al., 2019; Torii et al., 2015). The classical image retrieval task aims to identify all images depicting the same content as the query photo (Philbin et al., 2007, 2010; Sivic & Zisserman, 2003; Tolias et al., 2016). In contrast, place recognition requires only a single relevant image among the top-n retrieved images (Torii et al., 2015). In the context of structureless visual localization, place recognition approaches can be considered as pose approximation methods, which approximate the pose of the query by the poses of the top-retrieved images.

### Structure-based Localization

Structure-based methods are characterized by using a 3D model of the environment as a scene representation.

**Feature matching-based.** Early visual localization approaches based on feature matching represented the scene via Structure-from-Motion point clouds (Arth et al., 2009; Choudhary & Narayanan, 2012; Irschara et al., 2009; Li et al., 2010, 2012; Sattler et al., 2011, 2012, 2012; Se et al., 2002). Each 3D point was triangulated from features extracted from the database images used to build the SfM model. Thus, each point can be associated with the descriptors of its corresponding local image features. As a result, 2D-3D matches can be established by comparing feature descriptors extracted from the query image to descriptors associated with the 3D points (Choudhary & Narayanan, 2012; Li et al., 2010, 2012; Sattler et al., 2011, 2012; Se et al., 2002). The resulting 2D-3D correspondences *x*-*X* are then used to estimate the camera pose (rotation matrix *R* and translation vector *t*) of the query image by applying a PnP solver, typically a P3P solver for calibrated cameras (Ding et al., 2023; Fischler & Bolles, 1981; Haralick et al., 1994; Persson & Nordberg, 2018), inside RANSAC (Baráth et al., 2019; Chum & Matas, 2008; Fischler & Bolles, 1981; Lebeda et al., 2012). PnP estimates the pose by solving a system of equations based on the perspective projection equation

$$\begin{aligned} \lambda \begin{bmatrix} x \\ 1 \end{bmatrix} = K \begin{bmatrix} R&t \end{bmatrix} \begin{bmatrix} X \\ 1 \end{bmatrix}, \end{aligned}$$

where *K* is the calibration matrix and $\lambda$ is the projective depth scalar.

While such **direct methods** based on directly matching query and 3D point descriptors can run on mobile devices (Arth et al., 2009; Lynen et al., 2015), they struggle to scale to larger scenes (Li et al., 2012). This is due to ambiguities in local appearance arising in larger and more complicated scenes: At scale, it is likely that there are many 3D points with similar descriptors, making it hard to identify the corresponding 3D point for a given query feature (Li et al., 2012). These ambiguities can be resolved either by accepting more incorrect matches and filtering them out through geometric reasoning Svärm et al., 2017; (Zeisl et al., 2015), or by using image retrieval to restrict matching to subparts of the 3D model (Humenberger et al., 2022; Irschara et al., 2009; Sarlin et al., 2019, 2018; Sattler et al., 2015, 2012; Taira et al., 2019). The latter approaches are known as **hierarchical methods**, which only match query features against 3D points visible in the top-retrieved database images and constitute the current state of the art.

Although SfM point clouds are the dominant scene representation for feature-based methods, other representations, including meshes (Panek et al., 2022, 2023), Neural Radiance or Feature Fields (Chen et al., 2023; Liu et al., 2023; Zhou et al., 2024), and 3D Gaussian Splats (Matteo et al., 2025), are used. Still, these approaches are based on establishing 2D-3D matches using local features.

**Scene coordinate regression.** Rather than relying on descriptor matching to establish 2D-3D correspondences, scene coordinate regression methods train machine learning models to directly regress the corresponding 3D point position for a given input patch (Brachmann & Rother, 2018; Brachmann et al., 2017, 2023; Brachmann & Rother, 2020; Budvytis et al., 2019; Cavallari et al., 2019, 2019; Dong et al., 2022; Guzman-Rivera et al., 2014; Li et al., 2020; Massiceti et al., 2017; Shotton et al., 2013; Tang et al., 2021; Valentin et al., 2015; Wang et al., 2023). While early approaches used random forests (Guzman-Rivera et al., 2014; Massiceti et al., 2017; Shotton et al., 2013; Valentin et al., 2015), recent methods use neural networks. As for feature-based methods, the resulting 2D-3D correspondences are then used for RANSAC-based pose estimation. Whether scene coordinate regressors or feature-based approaches are more accurate is still an open question (Brachmann et al., 2021).

**Camera pose regression relative to 3D models.** Both feature-based methods and scene coordinate regressors establish 2D-3D matches for pose estimation. Given that an initial coarse pose estimate is typically available, *e.g.*, obtained via image retrieval, an alternative approach to structure-based localization is pose refinement. Such methods iteratively compare the query image against a rendering of a 3D model from the current pose estimate (Sarlin et al. 2021; Pietrantoni et al. 2023, 2024; Trivigno et al. 2024; Lin et al. 2020; Chen et al. 2023; Sun et al. 2023; Botashev et al. 2024; Zeller 2024; Liu et al. 2024; Von Stumberg et al. 2020a, b. The pose is then adjusted to reduce the difference between the image and the rendering. In terms of pose accuracy and robustness, such regression approaches are inferior to hierarchical feature-based methods.

### Structureless Localization

Structureless methods do not store any kind of 3D structure, but rather represent the scene with a set of reference images with known camera poses. Storing only images and poses has the advantage of significantly simpler representation update step relative to the structure-based methods. The update consists of simply adding new or removing outdated posed images and updating the retrieval index. We compare the update steps for the two sets of methods in Table 9.

**Pose approximation.** Given a database of images with known camera poses, the pose of a query image can be approximated efficiently through the pose of the top-retrieved image (Torii et al., 2015). Better approximations can be obtained by interpolating the poses of the top-n retrieved images (Sattler et al., 2019; Torii et al., 2015). (Thoma et al., 2020) proposed to learn descriptors for image retrieval such that distances in the descriptor space are proportional to pose similarity. The resulting descriptors enable more accurate pose approximation. Still, the pose quality is significantly below the requirements of precise visual localization. Hence, we do not evaluate pose approximation approaches in this work.

**Pose triangulation.** A more precise query pose can be obtained from the relative poses between the query and the top-retrieved database images (Dong et al., 2023; Laskar et al., 2017; Zhang & Kosecka, 2006; Zhou et al., 2019). Pairwise relative poses, *e.g.*, computed by estimating an essential matrix or homography, only provide the rotation $R_\text {i}$ and direction of the relative translation $t_\text {uts~i}$, but not its magnitude. Given that the poses of the database images are known, the position of the query photo can be triangulated from two or more relative translation directions (Laskar et al., 2017; Zhang & Kosecka, 2006; Zhou et al., 2019). The rays used for the triangulation can be defined as

$$\begin{aligned} C_\text {ref~i} + \gamma _\text {i}R^\top _\text {ref~i} R_\text {i}t_\text {uts~i}, \end{aligned}$$

where $C_\text {ref~i}$ is known camera center of a reference camera (from $\text {i}$-th query-reference pair), $R_\text {ref~i}$ is its rotation matrix and $\gamma _\text {i}$ is the unknown translation scale. Once we have the rays, we can triangulate the query camera center, *e.g.*, using the DLT (Direct Linear Transform) method (Hartley & Zisserman, 2001).

Inspired by global SfM approaches (Cui & Tan, 2015; Pan et al., 2024; Zhu et al., 2018), LazyLoc (Dong et al., 2023) further improves pose accuracy by adding rotation and translation averaging stages followed by a post optimization that jointly optimizes the query camera pose and the 3D points triangulated from 2D feature tracks. In this work, we consider both a “standard" pose triangulation approach (Zhou et al., 2019) and LazyLoc (Dong et al., 2023).

**Semi-generalized relative pose estimation.** The scale of the translation can be recovered when computing the pose of the query *w.r.t.* two or more database photos (Zheng & Wu, 2015), *i.e.*, when computing a semi-generalized relative pose rather than the relative pose between two images. Given the known poses of the database images, the relative pose of the query can then be directly translated into its absolute pose. The term “semi-generalized” refers to the combination of a generalized reference camera (a collection of rays belonging to multiple pinhole reference cameras) and a single pinhole query camera. Zheng and Wu (2015) derive multiple solvers for computing the semi-generalized relative pose between a query and two database images, where the relative pose between the database photos is known. The solvers differ based on the number of correspondences between the query and the other two images. However, all of the solvers except one are too slow to be used in practice. The remaining solver assumes 5 point correspondences between the query and one of the database images and a single correspondence between the query and the other database image. The 5 matches are used to compute the relative pose by estimating the essential matrix *E*, which can be done highly efficiently (Nistér 2004a). Each of the estimated essential matrices ((Nistér 2004a) gives up to 10 possible solutions) can be decomposed into 2 rotations (in opposite directions) *R* and a single up-to-scale translation $t_\text {uts}$. The full translation can be written as

$$\begin{aligned} t = R C_\text {ref1} + \gamma t_\text {uts}, \end{aligned}$$

where $C_\text {ref1}$ is camera center of the first sampled reference image. The remaining point correspondence $\hat{x}_\text {q}$ - $\hat{x}_\text {ref2}$ (in normalized coordinates $\hat{x} = K^{-1}x$) are then used to estimate the scale of the translation $\gamma$ by solving

$$\begin{aligned} R (C_\text {ref~2} - C_\text {ref~1} + \lambda _\text {2} \hat{x}_\text {ref~2}) + \gamma t_\text {uts} = \lambda _\text {q} \hat{x}_\text {q}. \end{aligned}$$

The solver is known as the E5+1 solver. We use a method that applies the solver inside a RANSAC loop as one of our baselines. As our experiments show, this approach is very competitive. Interestingly, it has not been used as a baseline in other works on structureless localization such as Laskar et al. (2017); Dong et al. (2023); Zhou et al. (2019).

Bhayani et al. (2021) shows that more efficient solvers for the setting described above can be derived by assuming that the scene is locally planar. These solvers are based on estimating homographies, hence (Bhayani et al., 2021) solve a semi-generalized homography estimation problem. In our experiments, we only evaluate a method build around the E5+1 solver due to its efficiency and easy implementation.

**Constructing SfM models on the fly.** A common approach to pose triangulation and semi-generalized relative pose estimation is to establish 2D-2D correspondences between the query and the top-retrieved database images. Implicitly, these matches define point correspondences between the retrieved database images. Together with the known poses of the database images, these correspondences can be used to triangulate 3D points. This results in 2D-3D matches for the query, which can then be used for pose estimation. As our experiments show, approaches that build such local SfM models on the fly (Humenberger et al., 2022; Pion et al., 2020; Torii et al., 2021) lead to the best pose accuracy among all tested structureless localization strategies. Their downside is the computational overhead caused by triangulation.

**Absolute pose regression.** The structureless localization approaches discussed above, with the exception of pose approximation methods, all establish 2D-2D feature matches between the query and the database images. These matches in turn are used to explicitly estimate the geometric relation between the photos. An alternative approach is to train a neural network to directly regress the pose of the query image (Brahmbhatt et al., 2018; Kendall & Cipolla, 2017; Kendall et al., 2015; Moreau et al., 2021; Shavit et al., 2021; Walch et al., 2017). However, as shown in Sattler et al. (2019), most of these absolute pose regression approaches are not significantly more accurate than pose approximation methods. These methods implicitly store the scene through the weights of a neural network. As such, updating the scene representation is non-trivial and will require fine-tuning the network. For these reasons, we do not consider absolute pose regression approaches in our experimental comparison.

**Relative pose regression.** Rather than regressing the absolute pose of a single image, relative pose regression approaches regress the relative pose between two input images (potentially including the scale of the translation) (Balntas et al., 2018; Ding et al., 2019; Dong et al., 2024; Laskar et al., 2017; Leroy et al., 2024; Ng et al., 2022; Wang et al., 2025, 2024; Zhou et al., 2019). In the context of structureless localization, relative pose regressors can thus be used instead of explicit geometric reasoning from feature matches. Although initial approaches were not significantly better than pose approximation algorithms (Sattler et al., 2019), recent methods (Dong et al., 2024; Leroy et al., 2024; Wang et al., 2025, 2024) are at least competitive with classical approaches. Under challenging conditions, especially if there is little visual overlap between images, they can significantly outperform classical approaches. Thus, we include the recent pose regression-based approaches (Dong et al., 2024; Leroy et al., 2024) in our evaluation.

## Selected Methods

This paper aims at understanding the performance of existing structureless visual localization approaches by comparing them through extensive experiments. In the following, we discuss the methods we selected for our comparison, grouped based on the family of approaches they belong to.

**Pose triangulation.** We evaluate two pose triangulation approaches: *Localization from essential matrices* (Zhou et al., 2019) (*Ess. mat.*) computes the relative poses between the query and the retrieved database images using the well-known 5-point algorithm (Nistér 2004a) (further dubbed as 5Pt) inside a RANSAC (Fischler & Bolles, 1981; Lebeda et al., 2012) loop. The final orientation of the query image is computed via averaging the relative rotations. The camera position is estimated by triangulation using the estimated translation directions. To evaluate this method, we reimplemented the functionality of the code provided with the original publication (Zhou et al., 2019).

We further evaluate a variant of *Ess. mat.* that uses a 3-point solver (Ding et al., 2025) instead of the 5-point solver to compute relative poses. The 3-point solver uses monocular depth predictions to compute the relative pose from fewer 2D-2D matches, making it more suitable for scenarios with low inlier ratios. The 3-point solver is similarly accurate as the 5-point solver when using reasonably accurate depth maps (Ding et al., 2025). We denote the resulting approach as *Ess. mat. (3Pt+depth)*. We use the solver implementation of the authors of Ding et al. (2025) together with our reimplementation of the *Ess. mat.* method.

*LazyLoc* (Dong et al., 2023) also uses the 5-point algorithm (Nistér 2004a) to obtain relative poses between the retrieved and the query image. The query pose is then computed using robust motion averaging with outlier rejection, followed by query pose optimization based on 2D-3D matches. For the latter, 3D points are triangulated from 2D-2D matches between the retrieved database images. The pose is then optimized via least-squares minimization of reprojection errors. The used implementation was kindly provided by the authors of Dong et al. (2023).

We selected *Ess. mat.* as an example of a rather straightforward pose triangulation approach, while *LazyLoc* represents the current state-of-the-art in terms of pose triangulation.

**Semi-generalized relative pose estimation.** As mentioned above, we build a localization system around the E5+1 solver (Zheng & Wu, 2015). The solver first estimates relative pose between the query image and one database image from 5 2D-2D correspondences using the 5-point solver (Nistér 2004b). One additional 2D-2D match between the query and another retrieved image is then used to recover the scale of the translation based on the known poses of the two database images. We apply the solver within RANSAC with local optimization (Lebeda et al., 2012). Local optimization is performed by least squares minimization of Sampson errors (Hartley & Zisserman, 2001) starting from poses estimated by the E5+1 solver. We use the implementation provided by PoseLib (Larsson, 2020) and refer to the approach as *E5+1*.

As for *Ess. mat.*, we also evaluate a variant of *E5+1* that uses the 3-point solver from Ding et al. (2025) instead of the 5-point solver to compute relative poses. We denote this approach as *E3+1*.

**SfM on the fly.** We evaluate two variants of an SfM on-the-fly pipeline. Both use 2D-2D matches between the query and the database images to obtain 2D-2D correspondences between the database photos.^3^ In both cases, these 2D-2D matches are used to triangulate 3D points. In turn, these points define 2D-3D matches for the query image, which are then used for absolute pose estimation with a P3P solver (Ding et al., 2023) inside RANSAC with local optimization. Given that we build local 3D models on demand, *i.e.*, based on the retrieved database images, and do not construct and maintain a single global model, we refer to both methods as *Local triangulation*.

The first variant uses all the database images retrieved for the single query image for triangulation (and is hence denoted as *Local triangulation - all*). Each feature in a query image defines a track containing feature keypoints found in the retrieved database images. Triangulation is then performed for every keypoint in the track within RANSAC, selecting the 3D point with the highest number of inliers in terms of a given reprojection error threshold.

The second variant, denoted as *Local triangulation - pairs*, considers pairs of database images for triangulation and pose estimation. For each potential pair of retrieved database images, it triangulates 3D points and estimates the query pose from the resulting 2D-3D matches. The final query pose is the one with the largest number of inliers.

For our experiments, we use both features based on sparse keypoint detections (DeTone et al., 2017; Zhao et al., 2023, 2022) and dense feature matchers (Edstedt et al., 2024; Leroy et al., 2024). The latter obtain 2D-2D correspondences by matching densely extracted features between two images. As a result, there are no repeatable keypoint detections between image pairs (Sun et al., 2021; Zhou et al., 2020). In order to be able to form tracks for dense matchers, matches having keypoints with mutually nearest coordinates in the query image (up to a distance threshold) are established as matches between the reference images. We used a distance threshold of 5 px in our experiments selected based on grid search. We implemented the pipelines using the point triangulation method from OpenCV (Bradski, 2000) and the P3P solver from PoseLib (Larsson, 2020; Persson & Nordberg, 2018).

**Regressor-based methods.**

**Fig. 2:**
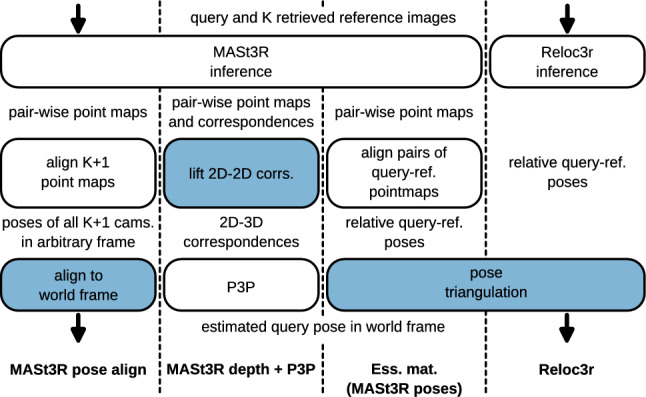
An overview of regressor-based pipelines

We evaluate multiple regression-based approaches (Dong et al., 2024; Leroy et al., 2024). An overview of the four pipelines presented below is presented in Fig. 2. The first method is built around the MASt3R (Leroy et al., 2024) foundation model.

MASt3R does not directly regress a relative pose, but a pair of pointmaps (one for each image), which can be aligned to produce a relative pose estimate. Given the query and a number of retrieved database images, we use MASt3R to build a local 3D model. To this end, MASt3R first regresses pairwise depth maps for all query-reference and reference-reference pairs (essentially using the DUSt3R (Wang et al., 2024) approach), which are subsequently aligned, followed by optimizing all the resulting camera poses. We then align this local reconstruction with the known poses of the database images using the Kabsch-Umeyama alignment (Kabsch, 1976; Umeyama, 1991) in two stages: the first stage only uses the camera positions for alignment, thus aligning the camera positions and recovering the scale of the local reconstruction.

In the case that the camera centers of the database images are (nearly) collinear, the alignment is ambiguous, as it is defined up to a rotation around the line containing the camera centers. We thus add a single 3D point on the optical axis of every reference image, one length unit from the camera center. We then recompute the alignment using the database image positions and the additional points.^4^ During our experiments, we observed that if there is a wrongly retrieved reference image without any overlap with query or other retrieved images, the optimizer still generates an arbitrary pose for it. As the wrong pose can then skew the global alignment, we want to prevent the use of such images. Therefore, the whole process of regression, optimization, and alignment is performed using a randomly sampled subset of the retrieved reference images and repeated multiple times. We also filter out all retrieved images that don’t have enough correspondences with query. The final pose estimate is selected on the basis of the number of inliers in terms of a given epipolar error threshold. We use the depth map regressor, the matcher, and the camera optimizer implemented within the MASt3R codebase together with our implementation of the alignment of the camera pose to the world frame. We denote this MASt3R-based localization approach as *MASt3R pose align*.

Note that the *MASt3R pose align* approach is suboptimal in the sense that the MASt3R implementation released by the authors of Leroy et al. (2024) cannot make use of known intrinsics and camera poses of the database images. However, to the best of our knowledge, the same limitation applies to all other 3D reconstruction approaches based on the relative pose regression (Dong et al., 2024; Wang et al., 2025, 2024). In all cases, adjusting the implementation is highly non-trivial, and we did not attempt the modification. Rather, we see the *MASt3R pose align* approach as a way to measure how well existing relative pose-based approaches work out of the box.

Based on the MASt3R paper (Leroy et al., 2024), the depth regressor was trained to perform metric-scale predictions. If the depth maps are metric, they can be used to lift the 2D-2D correspondences to 3D. The resulting 2D-3D matches can be used for pose estimation with P3P (Persson & Nordberg, 2018). We refer to this method as *MASt3R depth + P3P*.

Another approach we tested is using the pairwise relative poses generated by MASt3R with the depth map alignment and estimating the query camera pose using the *Ess. mat.* method. This approach is further referred to as *Ess. mat. (MASt3R poses)*.

The last approach we evaluate is *Reloc3r* (Dong et al., 2024). *Reloc3r* uses a neural network to regress relative camera poses between query-database image pairs. The absolute pose of the query image is then obtained via pose triangulation: The query image’s orientation is obtained by averaging the rotation matrices predicted from the relative poses to the database images and the known poses of the database images. The query’s camera position is computed using triangulation via the relative translation directions. The evaluation was performed using the implementation provided by the authors of the paper.

## Experimental Evaluation

**Datasets.** We evaluate the structureless visual localization approaches discussed above on multiple large datasets commonly used to benchmark visual localization algorithms: Aachen Day-Night v1.1 (Sattler et al., 2018, 2012; Zhang et al., 2020) is an outdoor dataset that captures the historic center of Aachen, containing time of day and seasonal changes. Extended CMU Seasons (Badino et al., 2011; Sattler et al., 2018) is an outdoor dataset with seasonal changes with multiple urban, suburban, and park scenes captured from a moving car. NAVER LABS Large-scale Localization Datasets in Crowded Indoor Spaces (Lee et al., 2021) (hereinafter referred to as NAVER datasets) consist of multiple scenes in shopping malls and a large metro station. For Aachen Day-Night we use undistorted reference images resized so that the longer side has a maximum of 800 pixels. For all the other datasets, we use the original unaltered images.

**Evaluation protocol.** We follow common practice for the datasets and report the percentage of query images that are localized within specific error thresholds (Sattler et al., 2018). *I.e.*, we report the percentage of query images localized within a *X*cm position error and a $Y^\circ$ rotation error of the corresponding ground-truth poses. We use the Long-Term Visual Localization benchmark website (Sattler et al., 2018) for obtaining the measures.

**Fig. 3:**
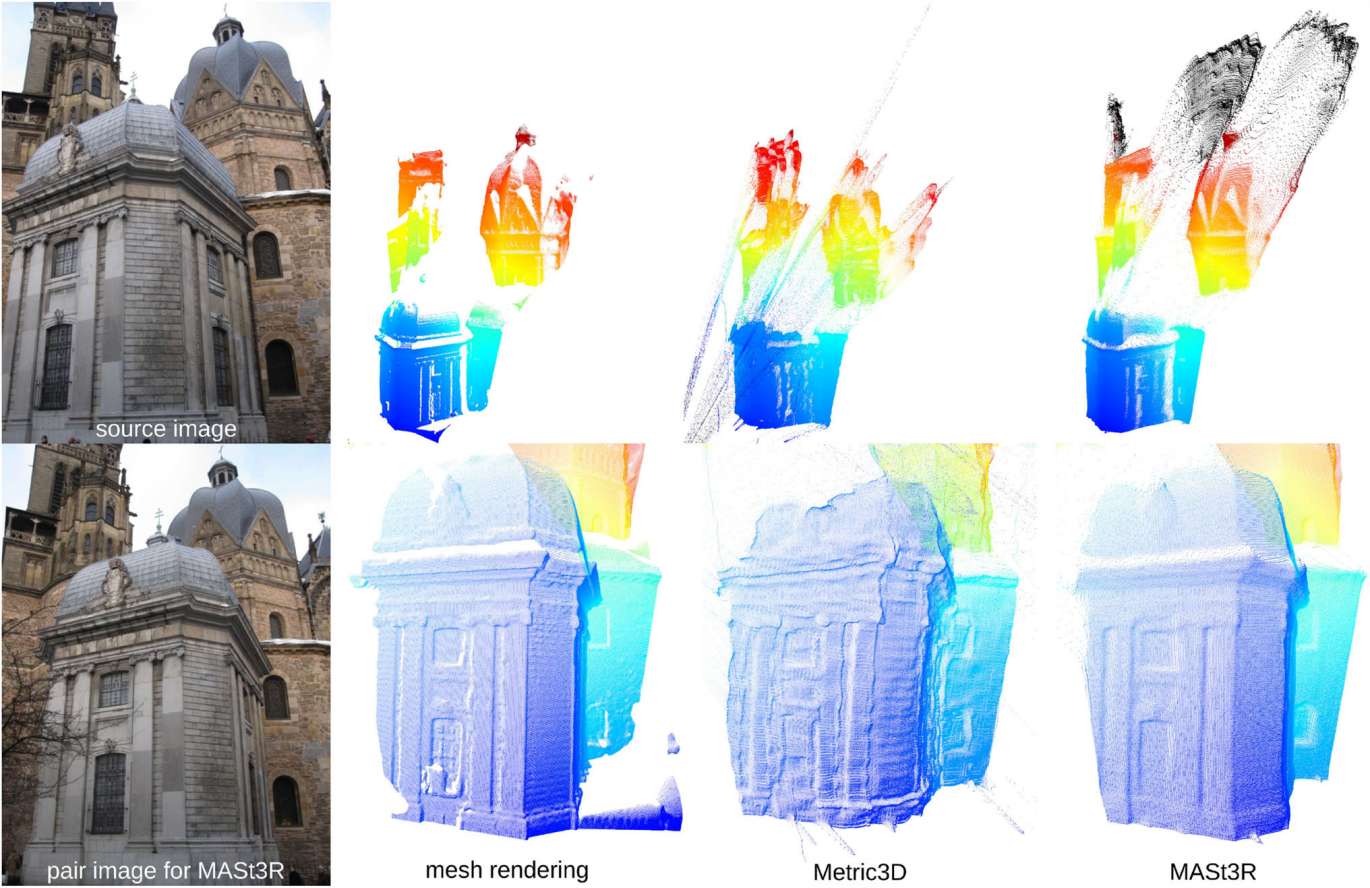
Comparison of depth maps from different sources - on the top left is the source image. The corresponding source camera was used for the rendering of a mesh model (AC-14 model from MeshLoc (Panek et al., 2022)). The source image together with its focal length is the sole input into the Metric3D v2 (Hu et al., 2024; Yin et al., 2023) monocular depth estimator. As MASt3R (Leroy et al., 2024; Wang et al., 2024) is a stereo model, it also uses a second image (shown in the bottom left) to predict the 3D geometry. MASt3R performs the prediction without any knowledge about the camera parameters. Both Metric3D and MASt3R depth maps were aligned (in scale and shift) to the mesh depth map for easier comparability, while they are used in their raw unscaled form in the experiments

**Implementation details.** All methods assume that a set of reference images with known poses and camera intrinsics is given. All baselines perform an initial image retrieval step using an image-level descriptor. Based on prior experience and preliminary experiments, we use the learned EigenPlaces descriptor (Berton et al., 2023). Most of the selected methods use 2D-2D feature matches between the query and the retrieved set of reference images to compute the pose. We evaluate two sparse local feature extractors, namely SuperPoint (DeTone et al., 2017) and ALIKED (Zhao et al., 2023, 2022), in combination with the LightGlue (Lindenberger et al., 2023) matcher. In addition, we also use two dense matchers, namely RoMa (Edstedt et al., 2024) and MASt3R (Leroy et al., 2024; Wang et al., 2024). For RoMa, we use the "outdoor" model for the Aachen Day-Night v1.1 and Extended CMU Seasons datasets and the "indoor" model for the NAVER datasets. In case of matching with MASt3R, we use only the coarse matching stage to keep the evaluation time within a reasonable scale.

For the *Local triangulation* methods, we use a 2px reprojection error threshold for SuperPoint and ALIKED features, and a 8px threshold for RoMa and MASt3R. These values were selected based on a grid search performed on thresholds of 1px, 2px, 4px, 8px and 16px on the Aachen Day-Night v1.1 dataset (Sattler et al., 2018, 2012; Zhang et al., 2020).

For *MASt3R pose align*, we sample a subset of 3 retrieved reference images and iterate the pose estimation 10 times. Only reference images with more than 50 correspondences with the query are used, as a wrongly retrieved image without an overlap with the query can significantly skew the resulting pose estimate. We use the two-stage optimization implemented in MASt3R (Leroy et al., 2024). The first stage minimizes 3D point map distances while optimizing only the cameras. The second stage minimizes reprojection errors while also changing the point maps. We use 300 iterations for each stage, and learning rates of 0.2, respectively, 0.02 for the first, respectively, second stage.

For inlier counting, we use reprojection and epipolar error thresholds of 12 px. For the *E5+1*, *E3+1* and local triangulation methods, we use a locally optimized RANSAC with a minimum of 1000 and a maximum of 100,000 iterations.

We use two state-of-the-art methods for depth prediction. The monocular metric depth estimator Metric3D v2 (Hu et al., 2024; Yin et al., 2023) and the stereo geometry regressor from MASt3R (Wang et al., 2024). We show a qualitative sample of the generated depth maps in Fig. 3. Both estimated depth maps were aligned in global scale and shift to a depth map rendered from a mesh.^5^ Although the Metric3D depth map is not very accurate in detail and contains upsampling artifacts on depth discontinuities, it is able to recover the global scale relatively well. For the presented image, the monocular depth was scaled down by a factor of 0.85 and shifted by +1.54 m during the alignment. The MASt3R model is capable of reconstructing the geometry in remarkable detail, but its ability to estimate the scale of the scene is very poor. The presented sample was scaled up by a factor of 15.83 and shifted by -0.10 m. The two depth predictors performed similarly for other images from the Aachen v1.1 dataset.

**Fig. 4:**
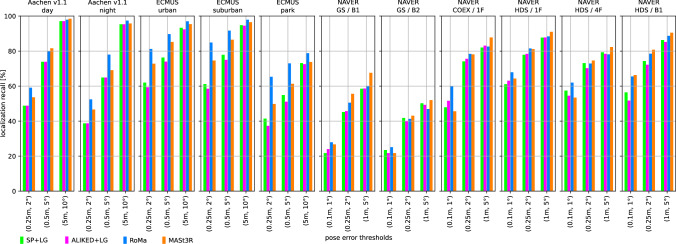
Localization results for the *Ess. mat. (5Pt)* approach for different features. We report localization recalls (higher is better) on the Y-axis at multiple pose thresholds (X-axis). For the outdoor scenes, the best results are obtained with the RoMa matcher. For the indoor scenes, the MASt3R matcher performs best for the coarser thresholds

**Fig. 5:**
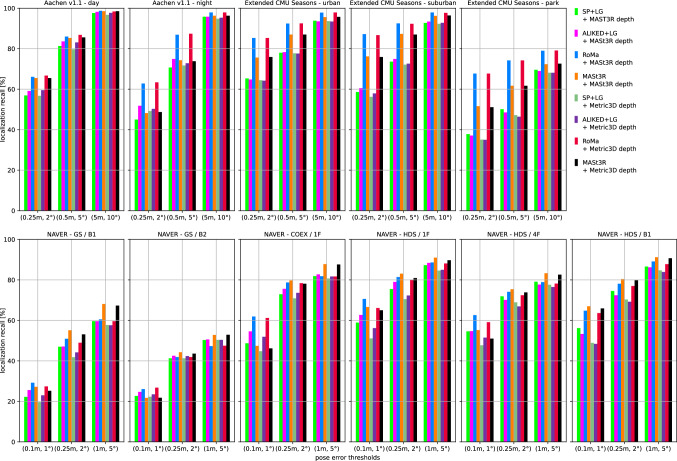
Localization results for the *Ess. mat. (3Pt + depth)* approach for different features and monocular depth predictors. We report localization recalls (higher is better) on the Y-axis at multiple pose thresholds (X-axis). For most scenes, the choice of the depth predictor is not critical. For outdoor scenes, RoMa yields the best results. For indoor scenes, MASt3R leads to the highest pose accuracy in most cases

**Fig. 6:**
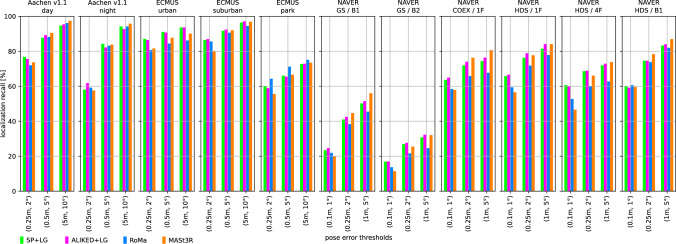
*LazyLoc* localization results for different features. We report localization recalls (higher is better) on the Y-axis at multiple pose thresholds (X-axis). There is no type of feature that performs best in all scenes. However, the MASt3R matcher performs well in general

**Fig. 7:**
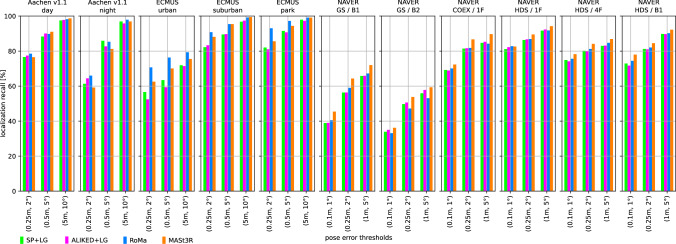
*E5+1* localization results for different features. We report localization recalls (higher is better) on the Y-axis at multiple pose thresholds (X-axis). For the outdoor scenes, the best results are typically obtained with the RoMa matcher. For the indoor scenes, the MASt3R matcher performs best for the coarser thresholds

**Fig. 8:**
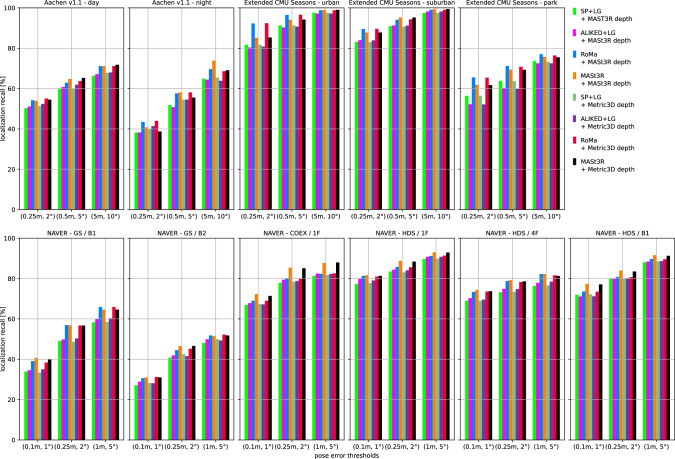
*E3+1* localization results for different features and depth predictors. We report localization recalls (higher is better) on the Y-axis at multiple pose thresholds (X-axis). The choice of the depth predictor is not critical

**Fig. 9:**
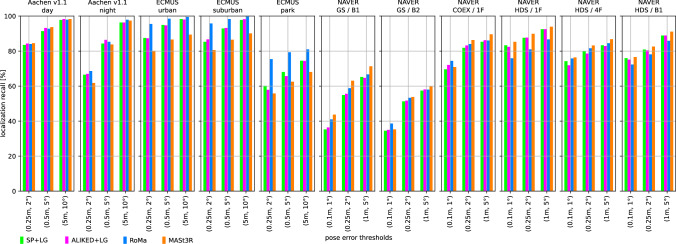
Localization results for the local 3D point triangulation from all retrieved images (*Local triangulation - all*) for different features. We report localization recalls (higher is better) on the Y-axis at multiple pose thresholds (X-axis)

**Fig. 10:**
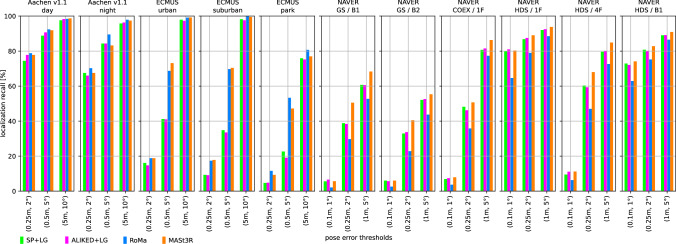
Localization results for the local 3D point triangulation from reference image pairs (*Local triangulation - pairs*) for different features. We report localization recalls (higher is better) on the Y-axis at multiple pose thresholds (X-axis)

### Ablation Studies

In a first set of experiments, we evaluate the impact of certain design choices (typically the type of features used and the type of depth maps used when applicable) on the performance of the various structureless localization methods. Sec. 4.2 then compares the best-performing versions of the different structureless methods with each other. Finally, Sec. 4.3 compares structureless methods with structure-based methods. Analysis of runtime and memory requirements is presented in Tabs. 6 and 7.

**Pose triangulation.** We present the results for *Ess. mat.* using the 5Pt solver in Fig. 4 and using the 3Pt+depth solver in Fig 5. As can be seen from Fig 5, the choice of the depth predictor does not seem to be critical, with both predictors performing similarly well.^6^ As can be seen from both figures, the RoMa matcher performs best for the outdoor scenes, while the MASt3R matcher typically yields the best accuracy in indoor scenes.

Fig. 6 shows results for *LazyLoc* when using different features. In contrast to the *Ess. mat.* approaches, there is no feature type that performs best in all scenes (or indoor or outdoor scenes). However, the MASt3R matcher gives good results in general and seems to be the feature of choice.

**Table 1:** Comparison of regression-based methods on Aachen Day-Night v1.1 (Sattler et al., 2018, 2012; Zhang et al., 2020). We use the top 10 images retrieved using the EigenPlaces (Berton et al., 2023) image-level descriptor. We report localization recalls (higher is better) at the pose thresholds of (0.25m, $2^{\circ }$) / (0.5m, $5^{\circ }$) / (5m, $10^{\circ }$).

Method	Matching	Day	Night
MASt3R pose align (Leroy et al., 2024; Wang et al., 2024)	MASt3R	9.6 / 39.6 / 88.6	9.4 / **37.2** / **88.0**
MASt3R depth (Leroy et al., 2024; Wang et al., 2024) + P3P (Persson & Nordberg, 2018)	MASt3R	0.1 / 1.1 / 37.7	0.0 / 0.5 / 33.0
Ess. mat. (MASt3R poses) (Leroy et al., 2024; Wang et al., 2024; Zhou et al., 2019)	MASt3R	**23.2** / **49.0** / **91.0**	**12.0** / 28.3 / 82.7
Reloc3r (Dong et al., 2024)	-	5.2 / 14.2 / 63.0	2.1 / 6.8 / 54.5

Best-performing methods are given in bold

**Table 2:** The best performing setup (matching method and depth map source) for each method evaluated on Aachen Day-Night v1.1 (Sattler et al., 2018, 2012; Zhang et al., 2020). We use the top 10 images retrieved using the EigenPlaces (Berton et al., 2023) image-level descriptor. We report localization recalls (higher is better) at pose thresholds of (0.25m, $2^{\circ }$) / (0.5m, $5^{\circ }$) / (5m, $10^{\circ }$)

Method	Matching	Depth	Day	Night
Ess. mat. (5Pt) (Zhou et al., 2019)	RoMa	-	59.1 / 80.0 / 97.9	52.4 / 78.0 / 97.4
Ess. mat. (3Pt+depth)	RoMa	Metric3D	66.7 / 86.8 / **98.4**	63.4 / 87.4 / **97.9**
LazyLoc (Dong et al., 2023)	MASt3R	-	73.7 / 90.5 / 97.5	57.6 / 83.8 / 95.8
E5+1	RoMa	-	78.4 / 89.8 / 97.8	65.4 / 84.8 / **97.9**
E3+1	RoMa	Metric3D	54.2 / 63.0 / 70.1	46.6 / 60.7 / 71.7
Local triang. - all	RoMa	-	**84.0** / **92.8** / 97.9	68.6 / 85.3 / **97.9**
Local triang. - pairs	RoMa	-	78.8 / 92.4 / **98.4**	**70.2** / **89.5** / **97.9**

Best-performing methods are given in bold

**Table 3:** The best performing setup (matching method and depth map source) for each method. Evaluated on Extended CMU Seasons (Badino et al., 2011; Sattler et al., 2018). We use the top 10 images retrieved using the EigenPlaces (Berton et al., 2023) image-level descriptor. We report localization recalls (higher is better) at the pose thresholds of (0.25m, $2^{\circ }$) / (0.5m, $5^{\circ }$) / (5m, $10^{\circ }$)

Method	Matching	Depth	Urban	Suburban	Park
Ess. mat. (5Pt) (Zhou et al., 2019)	RoMa	-	81.2 / 89.7 / 97.0	84.9 / 91.7 / 97.9	65.3 / 73.0 / 78.8
Ess. mat. (3Pt+depth)	RoMa	MASt3R	85.3 / 92.4 / 97.8	87.2 / 92.5 / 97.9	67.7 / 74.2 / 79.0
LazyLoc (Dong et al., 2023)	SP+LG	-	87.1 / 91.1 / 93.6	86.4 / 91.7 / 96.4	60.1 / 66.0 / 72.7
E5+1	RoMa	-	93.0 / 97.2 / 99.1	90.8 / 95.4 / 99.2	70.7 / 76.3 / 79.3
E3+1	RoMa	MASt3R	92.3 / 96.5 / 98.8	89.5 / 94.1 / 99.0	65.5 / 71.2 / 77.0
Local triang. - all	RoMa	-	**95.5** / **98.5** / **99.5**	**95.8** / **98.3** / 99.7	**75.4** / **79.3** / **81.0**
Local triang. - pairs	RoMa	-	18.8 / 68.7 / 99.1	17.4 / 69.7 / **99.8**	11.6 / 53.3 / 80.7

Best-performing methods are given in bold

**Table 4:** Benchmark on NAVER indoor localization datasets (Lee et al., 2021) Gangnam Station (GS) and COEX scenes. We use the top 10 images retrieved using the EigenPlaces (Berton et al., 2023) image-level descriptor. We report localization recalls (higher is better) at the pose thresholds of (0.1m, $1^{\circ }$) / (0.25m, $2^{\circ }$) / (1m, $5^{\circ }$).

Method	Matching	Depth	GS B1	GS B2	COEX 1F
Ess. mat. (5Pt) (Zhou et al., 2019)	MASt3R	-	26.7 / 55.6 / 67.6	21.7 / 43.1 / 52.0	45.6 / 78.1 / 87.8
Ess. mat. (3Pt+depth)	MASt3R	MASt3R	27.2 / 55.1 / 68.1	21.7 / 44.3 / 52.8	47.4 / 79.7 / 87.8
LazyLoc (Dong et al., 2023)	ALIKED+LG	-	24.6 / 42.5 / 51.5	17.0 / 27.6 / 32.3	64.9 / 74.0 / 76.4
E5+1	MASt3R	-	**45.4** / **64.3** / **72.0**	**36.2** / **53.8** / 59.3	**72.3** / **86.7** / **89.7**
E3+1	MASt3R	MASt3R	40.7 / 56.8 / 64.6	31.0 / 46.5 / 51.5	**72.3** / 85.4 / 87.7
Local triang. - all	MASt3R	-	43.7 / 63.1 / 71.3	35.3 / **53.8** / **59.8**	70.9 / 86.3 / 89.6
Local triang. - pairs	MASt3R	-	5.7 / 50.5 / 68.3	6.0 / 40.5 / 55.3	7.9 / 50.7 / 86.3

Best-performing methods are given in bold

**Table 5:** Benchmark on NAVER indoor localization datasets (Lee et al., 2021) Hyundai Department Store (HDS) scenes. We use the top 10 images retrieved using the EigenPlaces (Berton et al., 2023) image-level descriptor. We report localization recalls (higher is better) at the pose thresholds of (0.1m, $1^{\circ }$) / (0.25m, $2^{\circ }$) / (1m, $5^{\circ }$).

Method	Matching	Depth	HDS 1F	HDS 4F	HDS B1
Ess. mat. (5Pt) (Zhou et al., 2019)	MASt3R	-	64.3 / 81.1 / 91.0	53.4 / 74.6 / 82.3	66.3 / 80.8 / 90.5
Ess. mat. (3Pt+depth)	MASt3R	MASt3R	66.6 / 83.1 / 91.0	55.2 / 75.4 / 83.3	67.0 / 80.4 / 91.2
LazyLoc (Dong et al., 2023)	ALIKED+LG	-	66.6 / 78.9 / 84.1	59.8 / 68.9 / 72.9	59.2 / 74.6 / 84.0
E5+1	MASt3R	-	82.6 / **89.5** / **94.2**	**78.2** / **84.1** / **86.9**	**78.0** / **84.5** / **92.2**
E3+1	MASt3R	MASt3R	81.7 / 88.8 / 93.0	74.4 / 79.2 / 82.2	77.4 / 84.0 / 91.6
Local triang. - all	MASt3R	-	**85.3** / 89.9 / 93.9	76.4 / 83.2 / 86.8	76.6 / 82.6 / 91.1
Local triang. - pairs	MASt3R	-	80.3 / 89.0 / 93.8	11.2 / 68.0 / 84.9	74.1 / 82.8 / 90.9

Best-performing methods are given in bold

**Fig. 11:**
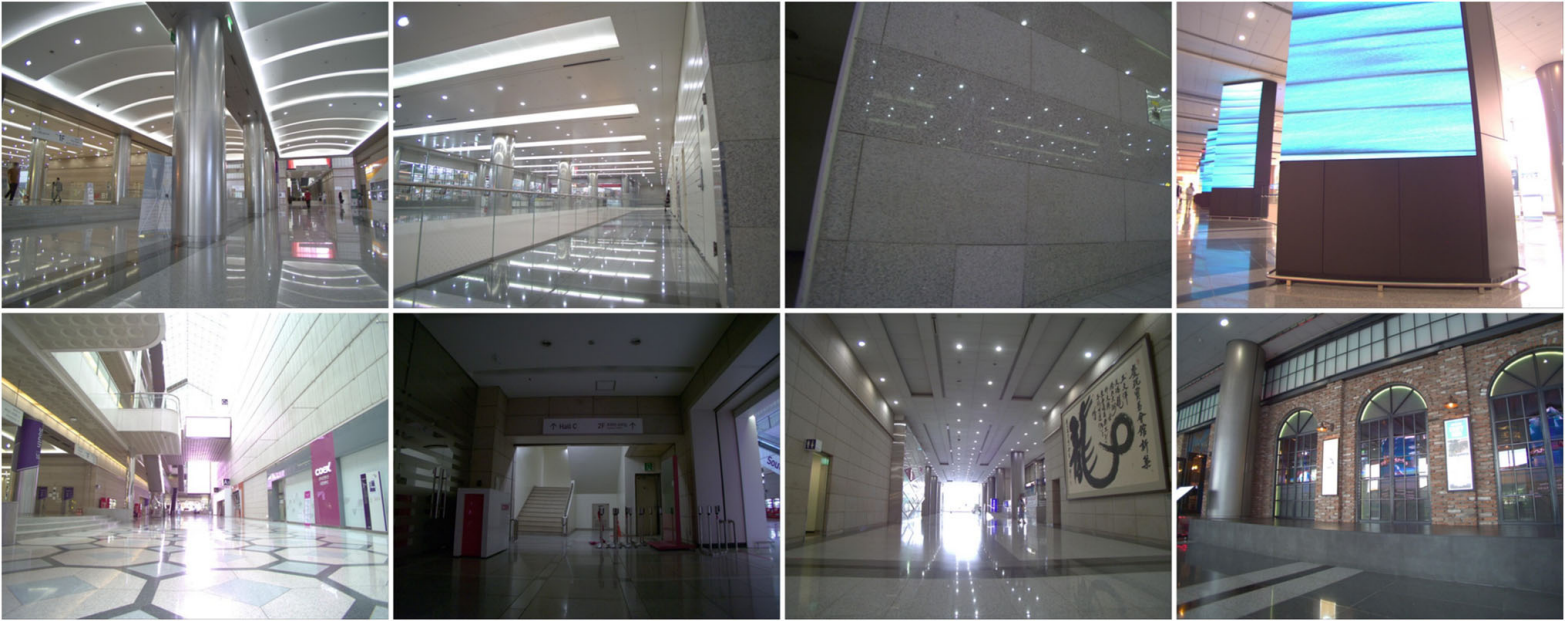
Comparison of the query images with high camera position error (top row) and the images with low camera position error (bottom row) in NAVER indoor localization dataset (Lee et al., 2021) COEX 1F scene. The former often contain repetitive patterns or other structures that complicate 3D point triangulation

**Semi-generalized relative pose estimation.** Figures 7 and 8 show results for the *E5+1* respectively the *E3+1* approach. For *E5+1*, the dense RoMA matcher often performs best in outdoor scenes while the dense MASt3R matcher performs best indoors. For *E3+1*, the RoMA matcher performs best on the Extended CMU Seasons dataset, while the MASt3R matcher works similarly well or better in all other scenes. As for *Ess. mat. (3Pt + depth)*, the choice of the monocular depth predictor is not critical. Note that, as for *Ess. mat. (3Pt + depth)*, the scale of the predicted depth maps is not important as the additional point correspondence with a second database image is used to recover the scale of the translation.

**SfM on the fly.** The results for the two local 3D point triangulation methods are shown in Fig. 9 respectively Fig 10. For the *Local triangulation - all* approach (Fig. 9), we observe that all features (except MASt3R) perform similarly well on Aachen Day-Night. For the Extended CMU Seasons dataset, RoMA clearly provides the best results, while MASt3R is typically the best for the indoor scenes. Interestingly, both SuperPoint and ALIKED outperform MASt3R on the Extended CMU dataset. In contrast, for *Local triangulation - pairs* (Fig. 10), both RoMa and MASt3R perform similarly to or better than SuperPoint and ALIKED. Interestingly, MASt3R performs best on Extended CMU when using pairs, while performing worse when using all images for triangulation. We speculate that some of the keypoint positions of MASt3R can be rather noisy. This negatively impacts the accuracy of the triangulated 3D points when using all images. When using pairs, there is a chance to obtain more accurate point positions by selecting two database images with consistent keypoints. In contrast, the other features benefit from using all images.

**Regressor-based methods.** Table 1 shows results for the four regressor-based methods on the Aachen Day-Night v1.1 (Sattler et al., 2018, 2012; Zhang et al., 2020) dataset. Compared to all previously evaluated approaches, all variants of pose regression-based methods perform significantly worse. The *Ess. mat. (MASt3R poses)* performs the best among these methods, but is still significantly less accurate than *Ess. mat. (5Pt)*. Even though the MASt3R depth regressor was trained to predict metric depth maps, our experiments show that the depths are far from metric. Thus, using them for lifting 2D-2D correspondence to 2D-3D matches, followed by P3P-RANSAC-based pose estimation, results in very inaccurate poses (see  *MASt3R depth + P3P* in Table 1). Our *MASt3R pose align* pipeline achieves better results than *Reloc3r*, but is still less accurate than *Ess. mat. (MASt3R poses)*.

Given the inaccurate pose estimates observed for the Aachen dataset, coupled with long run-times, we did not evaluate the pose regression-based approaches on other datasets.

**Discussion.** For most of the methods, RoMa (Edstedt et al., 2024) (with its outdoor model) usually performs the best for outdoor data sets (especially the Extended CMU Seasons dataset), while MASt3R (Leroy et al., 2024) typically performs better for indoor data sets. For indoor datasets, we observed that using RoMa’s outdoor model instead of its indoor model gives on average similar results.

Sparse features (SuperPoint and ALIKED) can achieve a similar or even better accuracy than dense matchers for some methods and scenes (*e.g.*, *LazyLoc* on most scenes and *Local triangulation* methods for the Aachen dataset). For other methods and certain scenes, they perform significantly worse, especially for *Local triangulation - pairs* on the Extended CMU Seasons dataset). Overall, dense matching methods often perform similar or better than sparse features. Still, they are not always the best choice.

The two depth estimators used for *Ess. mat. (3Pt+depth)* and *E3+1* lead to very similar pose accuracies, despite what we can see in Fig. 3. As observed above, both monocular depth estimators differ in the accuracy of their scale estimates. However, as also discussed above, the scale of the depth estimates is not used by either of the two methods. Still, Fig. 3 shows clear depth errors present in the Metric3D depth maps (Hu et al., 2024; Yin et al., 2023). This is likely due to two reasons: By applying the 3Pt solver (Ding et al., 2025) inside RANSAC, we can ignore regions with inaccurate depth estimates. At the same time, the initial poses obtained via the 3Pt solver are later refined. The refinement does not use the depth maps, thus allowing us to better handle inaccuracies.

### Comparing Structureless Localization Approaches

**Table 6:** Comparison of runtime and memory requirements, presenting the averaged measured values from Table 7. We assume that the pipelines start with a set of known reference image poses. The structure-based pipeline (Hloc) stores local and global features (and discards the images, which prevents switching to a different type of features later). In contrast, the structureless methods store only images and global features, extracting local features on the fly.

Method	Preprocessing (offline)	Localization (online)	Memory
$$\begin{array}{ll}\hbox {Hierarchical localization} \\ \hbox {(pre-extracted database features)}\end{array}$$	$$\begin{array}{ll}\text {N} \cdot \text {T}_\text {ext}\_\text {global} + \text {T}_\text {build}\_\text {ret} + \\ \text {N} \cdot \text {T}_\text {ext}\_\text {local} + \text {M} \cdot \text {T}_\text {match} + \\ \text {N} \cdot \text {T}_\text {build}\_\text {tracks} + \text {T}_\text {triang}\_\text {all}(\text {N})\end{array}$$	$$\begin{array}{ll}\text {T}_\text {ext}\_\text {global} + \text {T}_\text {ext}\_\text {local} + \text {T}_\text {ret} + \\ \text {K} \cdot \text {T}_\text {match} + \text {R} \cdot \text {T}_\text {abs}\_\text {pose}\end{array}$$	$$\begin{array}{ll}\text {N} \cdot \text {M}_\text {desc}\_\text {global} + \text {N} \cdot \text {M}_\text {idx} + \\ \text {N} \cdot \text {M}_\text {desc}\_\text {local} + \text {M}_\text {3D}\end{array}$$
structure-based	9 448.38 s	1 272.15 ms	5 948.01 MB
$$\begin{array}{ll}\hbox {Pose triangulation} \\ \hbox {(Ess. mat. (5Pt))} \end{array}$$	$$\begin{array}{ll}\text {N} \cdot \text {T}_\text {ext}\_\text {global} + \text {T}_\text {build}\_\text {ret}\end{array}$$	$$\begin{array}{ll}\text {T}_\text {ext}\_\text {global} + (\text {K}+1) \cdot \text {T}_\text {ext}\_\text {local} + \text {T}_\text {ret} + \\ \text {K} \cdot \text {T}_\text {match} + \text {K} \cdot \text {R} \cdot \text {T}_\text {rel}\_\text {pose} + \text {T}_\text {pos}\_\text {triang}\end{array}$$	$$\begin{array}{ll}\text {N} \cdot \text {M}_\text {image} + \\ \text {N} \cdot \text {M}_\text {desc}\_\text {global}\end{array}$$
structureless	913.93 s	2 655.40 ms	5 509.40 MB
$$\begin{array}{ll}\hbox {Semi-generalized rel. pose est.} \\ \hbox {(E5+1)}\end{array}$$	$$\begin{array}{ll}\text {N} \cdot \text {T}_\text {ext}\_\text {global} + \text {T}_\text {build}\_\text {ret}\end{array}$$	$$\begin{array}{ll}\text {T}_\text {ext}\_\text {global} + (\text {K}+1) \cdot \text {T}_\text {ext}\_\text {local} + \\ \text {T}_\text {ret} + \text {K} \cdot \text {T}_\text {match} + \text {R} \cdot \text {T}_\text {gen}\_\text {rel}\_\text {pose}\end{array}$$	$$\begin{array}{ll}\text {N} \cdot \text {M}_\text {image} + \\ \text {N} \cdot \text {M}_\text {desc}\_\text {global}\end{array}$$
structureless	913.93 s	2 168.19 ms	5 509.40 MB
$$\begin{array}{ll}\hbox {Local SfM} \\ \hbox {(Local triang. - all)}\end{array}$$	$$\begin{array}{ll}\text {N} \cdot \text {T}_\text {ext}\_\text {global} + \text {T}_\text {build}\_\text {ret}\end{array}$$	$$\begin{array}{ll}\text {T}_\text {ext}\_\text {global} + (\text {K}+1) \cdot \text {T}_\text {ext}\_\text {local} + \text {T}_\text {ret} + \\ \text {K} \cdot \text {T}_\text {match} + \text {T}_\text {triang}\_\text {all}(\text {K}) + \text {R} \cdot \text {T}_\text {abs}\_\text {pose}\end{array}$$	$$\begin{array}{ll}\text {N} \cdot \text {M}_\text {image} + \\ \text {N} \cdot \text {M}_\text {desc}\_\text {global}\end{array}$$
structureless	913.93 s	5 388.18 ms	5 509.40 MB
$$\begin{array}{ll}\hbox {Local SfM }\\ \hbox {(Local triang. - pairs)} \end{array}$$	$$\begin{array}{ll}\text {N} \cdot \text {T}_\text {ext}\_\text {global} + \text {T}_\text {build}\_\text {ret}\end{array}$$	$$\begin{array}{ll}\text {T}_\text {ext}\_\text {global} + (\text {K}+1) \cdot \text {T}_\text {ext}\_\text {local} + \text {T}_\text {ret} + \\ \text {K} \cdot \text {T}_\text {match} + \text {T}_\text {triang}\_\text {pairs}(\text {K}) + \text {R} \cdot \text {T}_\text {abs}\_\text {pose}\end{array}$$	$$\begin{array}{ll}\text {N} \cdot \text {M}_\text {image} + \\ \text {N} \cdot \text {M}_\text {desc}\_\text {global}\end{array}$$
structureless	913.93 s	3 050.08 ms	5 509.40 MB
$$\begin{array}{ll}\hbox {Rel. pose regression} \\ \hbox {(MASt3R pose align)}\end{array}$$	$$\begin{array}{ll}\text {N} \cdot \text {T}_\text {ext}\_\text {global} + \text {T}_\text {build}\_\text {ret}\end{array}$$	$$\begin{array}{ll}\text {T}_\text {ext}\_\text {global} + \text {T}_\text {ret} + {\text {R}_\text {M}}\text {K}_\text {M}(\text {K}_\text {M}+1) / 2 \cdot \text {T}_\text {MASt3R} + \\ \text {R}_\text {M} \cdot \text {T}_\text {align}\_\text {local}(\text {K}_\text {M}+1) + \text {R}_\text {M} \cdot \text {T}_\text {align}\_\text {world}\end{array}$$	$$\begin{array}{ll}\text {N} \cdot \text {M}_\text {image} + \\ \text {N} \cdot \text {M}_\text {desc}\_\text {global}\end{array}$$
structureless	913.93 s	237 597.86 ms	5 509.40 MB
$$\begin{array}{ll}\hbox {Rel. pose regression} \\ \hbox {(Ess. mat. (MASt3R poses))}\end{array}$$	$$\begin{array}{ll}\text {N} \cdot \text {T}_\text {ext}\_\text {global} + \text {T}_\text {build}\_\text {ret}\end{array}$$	$$\begin{array}{ll}\text {T}_\text {ext}\_\text {global} + \text {T}_\text {ret} + \text {K} \cdot \text {T}_\text {MASt3R} + \\ \text {T}_\text {pos}\_\text {triang}\end{array}$$	$$\begin{array}{ll}\text {N} \cdot \text {M}_\text {image} + \\ \text {N} \cdot \text {M}_\text {desc}\_\text {global}\end{array}$$
structureless	913.93 s	5 164.74 ms	5 509.40 MB

Used notation:

$\bullet$ N - number of reference images (6697)

$\bullet$ M - number of reference image pairs ($10 \cdot 6697$)

$\bullet$ K - number of retrieved reference images per single query image (10)

$\bullet$ R - number of RANSAC iterations (between 1 000 and 100 000 with early stopping)

$\bullet$ $\text {K}_\text {M}$ - size of the subset of retrieved images used for outlier filtering in *MASt3R pose align* (3)

$\bullet$ $\text {R}_\text {M}$ - number of loop iterations used for outlier filtering in *MASt3R pose align* (10)

$\bullet$ $\text {T}_\text {ext}\_\text {global}$ - time needed to extract global feature from a single image

$\bullet$ $\text {T}_\text {build}\_\text {ret}$ - time needed to build the retrieval index from scratch

$\bullet$ $\text {T}_\text {ret}$ - time needed for image retrieval for a single query

$\bullet$ $\text {T}_\text {ext}\_\text {local}$ - time needed to extract local features from a single image

$\bullet$ $\text {T}_\text {match}$ - time needed for local feature matching between a pair of images

$\bullet$ $\text {T}_\text {build}\_\text {tracks}$ - time needed to collect corresponding keypoints among images

$\bullet$ $\text {T}_\text {triang}\_\text {all}(\text {N})$ - time needed for triangulation from N reference images (using DLT)

$\bullet$ $\text {T}_\text {triang}\_\text {pairs}(\text {N})$ - time needed for triangulation from N reference images (using image pairs)

$\bullet$ $\text {T}_\text {abs}\_\text {pose}$ - time needed for a single RANSAC iteration with absolute pose solver

$\bullet$ $\text {T}_\text {gen}\_\text {rel}\_\text {pose}$ - time needed for a single RANSAC iteration with E5+1 pose solver

$\bullet$ $\text {T}_\text {rel}\_\text {pose}$ - time needed for a single RANSAC iterations with a relative pose solver

$\bullet$ $\text {T}_\text {pos}\_\text {triang}$ - time needed for position triangulation from a set of relative poses

$\bullet$ $\text {T}_\text {MASt3R}$ - time needed for MASt3R inference on a pair of images

$\bullet$ $\text {T}_\text {align}\_\text {local}(K+1)$ - time needed for alignment of reference images and the query based on pairwise pointmaps

$\bullet$ $\text {T}_\text {align}\_\text {world}$ - time needed for alignment of local reconstruction to the known reference camera poses

$\bullet$ $\text {M}_\text {desc}\_\text {global}$ - memory needed for global image descriptor for single reference image

$\bullet$ $\text {M}_\text {desc}\_\text {local}$ - memory needed for local image descriptors and keypoint coordinates for single reference image

$\bullet$ $\text {M}_\text {idx}$ - memory needed for associations between keypoints and 3D points for a single reference image

$\bullet$ $\text {M}_\text {image}$ - memory needed for a single reference image

$\bullet$ $\text {M}_\text {3D}$ - memory needed for the 3D model of the environment

**Table 7:** Runtime and memory requirements for individual pipeline blocks (as described in Table 6) on Aachen Day-Night v1.1. (Sattler et al., 2018, 2012; Zhang et al., 2020) dataset (with 6697 reference and 1040 query images). The reported total runtime of feature extraction is measured for the reference image set and the total retrieval and matching time is for matching between query images and their top-10 retrieved reference images (10400 pairs). The retrieval index build time corresponds to GPU-based FAISS (Douze et al., 2024) exact inverted file index on EigenPlaces descriptors. Note that the time measurements for pose estimation, pose triangulation and alignment operations are sums over whole RANSAC loop (running between 1 000 and 100 000 iterations $\text {R}$ with early stopping). The reported feature storage sizes correspond to the sizes of HDF5 files used by Hloc framework (Sarlin et al., 2019). The SfM model size is the size of COLMAP sparse reconstruction (in binary format) using SuperPoint+LightGlue matching on top-10 images retrieved with EigenPlaces. The experiments were performed using NVIDIA Tesla V100 SXM2 32 GB GPU and Intel Xeon E5-2698 v4 (2.20 GHz) CPU

Measured Quantity		Value Per Unit	Total
$\text {T}_\text {ext}\_\text {global}$	EigenPlaces	136.18 ms/image	912.01 s
$\text {T}_\text {build}\_\text {ret}$	EigenPlaces	0.29 ms/image	1.92 s
$\text {T}_\text {ext}\_\text {local}$	SuperPoint	79.09 ms/image	529.63 s
	ALIKED	80.64 ms/image	540.02 s
$\text {T}_\text {ret}$	EigenPlaces	120.19 ms/query	125.00 s
$\text {T}_\text {match}$	SP+LG	65.12 ms/pair	677.29 s
	ALIKED+LG	67.25 ms/pair	699.40 s
	RoMa	999.47 ms/pair	10 394.52 s
	MASt3R	855.07 ms/pair	8 892.70 s
$\text {T}_\text {MASt3R}$		466.01 ms/pair	9 460.02 s
$\text {T}_\text {build}\_\text {tracks}$	SP+LG	30.10 ms/pair	313.08 s
$\text {T}_\text {triang}\_\text {all}(\text {N})$	SP+LG	468.16 ms/image	3 135.29 s
$\text {T}_\text {triang}\_\text {all}(\text {K})$	SP+LG	3 325.13 ms/query	3 458.14 s
$\text {T}_\text {triang}\_\text {pairs}(\text {K})$	SP+LG	987.03 ms/query	1 026.51 s
$\text {R} \cdot \text {T}_\text {abs}\_\text {pose}$	P3P	285.49 ms/query	296.91 s
$\text {K} \cdot \text {R} \cdot \text {T}_\text {rel}\_\text {pose}$	5Pt	629.57 ms/query	654.75 s
	3Pt+depth	321.08 ms/query	333.92 s
$\text {R} \cdot \text {T}_\text {gen}\_\text {rel}\_\text {pose}$	E5+1	390.63 ms/query	406.25 s
	E3+1	476.13 ms/query	495.17 s
$\text {T}_\text {pos}\_\text {triang}$		248.27 ms/query	258.20 s
$\text {R}_\text {M} \cdot \text {T}_\text {align}\_\text {local}$		209.35 s/query	217 725.39 s
$\text {R}_\text {M} \cdot \text {T}_\text {align}\_\text {world}$		30.15 ms/query	31.36 s
$\text {M}_\text {image}$	max. 800 px	816.47 kB/image	5 467.89 MB
$\text {M}_\text {desc}\_\text {global}$	EigenPlaces	6.20 kB/image	41.51 MB
$\text {M}_\text {desc}\_\text {local}$	SuperPoint	827.41 kB/image	5 541.19 MB
	ALIKED	504.25 kB/image	3 376.98 MB
$\text {N} \cdot \text {M}_\text {idx} + \text {M}_\text {3D}$	COLMAP	-	365.31 MB

In the next set of experiments, we compare the different structureless localization approaches with each other. As discussed, we exclude pose regression-based approaches. For each method, we selected the best-performing setup (type of features and depth estimator) per dataset.

Table 2 shows results for the Aachen Day-Night v1.1 (Sattler et al., 2018, 2012; Zhang et al., 2020) dataset, Table 3 shows results for the Extended CMU Seasons (Badino et al., 2011; Sattler et al., 2018) dataset, and Table 4 and Table 5 show results for the NAVER indoor localization (Lee et al., 2021) datasets. In addition, Table 6 reports the average run times of the localization methods on the Aachen Day-Night v1.1 (Sattler et al., 2018, 2012; Zhang et al., 2020) dataset.

In terms of pose accuracy, methods that rely on more extensive geometric reasoning perform in general better: *Ess. mat. (5Pt)*, which uses pairwise relative poses obtained from 2D-2D matches, performs consistently worse than *Ess. mat. (3Pt+depth)*, which also uses depth maps for relative pose estimation. *LazyLoc* uses motion averaging and refinement based on 2D-3D matches, which can further boost performance. Interestingly, *LazyLoc* performs significantly worse than *Ess. mat. (3Pt+depth)* in some scenes (Aachen Night, Extended CMU Seasons park, NAVER GS B1 & B2, NAVER HDS 1F & B1). In some of these scenes, even *Ess. mat. (5Pt)* outperforms *LazyLoc*, indicating that the additional refinement steps might not be effective under all conditions. The results of the pipeline using the 5Pt algorithm with rotation averaging and camera center triangulation (*Ess. mat. - 5Pt*) are in line with the results reported in the literature on smaller datasets (Arnold et al., 2022; Dong et al., 2023).

*Ess. mat.* and *LazyLoc* first compute pairwise relative pose estimates with the retrieved database images, which are then fused into the final query pose prediction. In contrast, *E5+1* and *E3+1* directly compute the query pose *w.r.t.* multiple database images, which further improves performance. Unlike for *Ess. mat.*, using depth maps as part of the solver (*i.e.*, using the E3+1 instead of the E5+1 solver) can lead to inferior results. However, this behavior seems scene-dependent: The accuracy drop is most notable for the Aachen dataset, where the scene structure is often tens of meters away from the query image. In such a scenario, inaccuracies in the depth maps propagate to large pose errors. An interesting direction for future work is thus to automatically decide when to use monocular depth map predictions.

Compared to *E5+1* and *E3+1*, *Local triangulation* methods generate a local 3D model of the scene and use it to estimate the query pose. When using all pairs (*Local triangulation - all*), this approach performs the best on the outdoor scenes, while being slightly less accurate than *E5+1* on the indoor scenes. We observe a significant drop in pose accuracy when using only image pairs (*Local triangulation - pairs*) on the Extended CMU dataset and in some indoor scenes (Gangnam Station B1 and B2, COEX 1F, and Hyundai Department Store 4F), which might be caused by narrow corridors, view occlusion by pedestrians, repetitive geometric patterns and textures and reflective surfaces (see Fig. 11), making the triangulation from the image pairs more challenging compared to using all retrieved images.

In terms of pose accuracy, the *Local triangulation - all* and *E5+1* approaches are clearly the best choices. Taking run-times into account (*cf.* Table 6), the *E5+1* offers a better trade-off between pose accuracy and run-time. When run-times are a major concern, *LazyLoc* is a good alternative to these other two methods as it provides the fastest run-times while achieving reasonable pose accuracy on the outdoor datasets.

### Comparison with Structure-based Methods

**Table 8:** Comparison of the best structureless methods with state-of-the-art structure-based methods on Aachen Day-Night v1.1 (Sattler et al., 2018, 2012; Zhang et al., 2020). We use the top-10 and top-20 images retrieved using the EigenPlaces (Berton et al., 2023) image-level descriptor for all methods except *MASt3R vis. loc.*. We report localization recalls (higher is better) at the pose thresholds of (0.25m, $2^{\circ }$) / (0.5m, $5^{\circ }$) / (5m, $10^{\circ }$). The first three method are structure-based approaches.

method	matching	top-k	day	night
Hloc (Sarlin et al., 2019, 2020)	SP+LG	20	**88.1** / **95.4** / 99.0	71.7 / 89.0 / 97.9
		10	87.0 / 94.8 / 98.5	70.2 / 87.4 / 97.4
MeshLoc (Panek et al., 2022)	SP+LG	20	85.9 / 93.6 / 98.8	70.2 / 87.4 / 97.9
		10	84.2 / 92.5 / 98.5	70.2 / 85.9 / 96.9
MASt3R vis. loc. (Leroy et al., 2024)	R2D2 / MASt3R	20	83.4 / 95.3 / **99.4**	**76.4** / **91.6** / **100**
LazyLoc (Dong et al., 2023)	SP+LG	20	77.7 / 88.8 / 94.3	58.1 / 84.3 / 94.2
		10	76.8 / 87.7 / 94.7	58.1 / 84.3 / 94.2
E5+1	SP+LG	20	77.8 / 89.1 / 98.1	65.4 / 85.9 / 96.3
		10	76.6 / 88.3 / 97.5	61.3 / 85.9 / 96.9
Local triang. - all	SP+LG	20	86.7 / 93.8 / 98.3	67.5 / 85.3 / 97.4
		10	83.5 / 91.4 / 97.8	66.5 / 84.3 / 96.3

Best-performing methods are given in bold

This experiment compares the best-performing structureless methods with state-of-the-art structure-based methods: *Hloc* (Sarlin et al., 2019, 2020) is a hierarchical structure-based pipeline using a SfM point cloud as a scene representation. We evaluated Hloc with an SfM point cloud triangulated using the ground-truth reference camera poses. Each query image is matched against the top-10 respectively top-20 reference images retrieved using EigenPlaces (Berton et al., 2023) image-level descriptors. Feature extraction and matching were performed using SuperPoint (DeTone et al., 2017) and LightGlue (Lindenberger et al., 2023). *MeshLoc* (Panek et al., 2022) is a hierarchical pipeline that uses reference depth maps rendered from a triangular mesh to lift the 2D-2D query-reference correspondences to 2D-3D matches. Our evaluation uses the same RGB reference images as all the other methods. Only the depth maps are rendered from the AC-14 mesh, which in the original paper offered the best results. *MASt3R vis. loc.* is a hierarchical structure-based localization pipeline presented in Leroy et al. (2024). It uses a precomputed 3D point cloud, triangulated using R2D2 features matches (Revaud et al., 2019), which is projected into the reference images in order to lift the 2D-2D correspondences established with the MASt3R matcher between the query and the retrieved reference images to 2D-3D correspondences. The pose is then estimated using the P3P pose solver inside RANSAC.

Table 8 compares these structure-based methods against the best-performing structureless approaches from our previous experiments. As can be seen, Hloc still maintains the first place, with MeshLoc a little behind. The best of the structureless methods is *Local triang. - all*, being on par with MeshLoc on the day split of the dataset and a few percent behind on the night split. The other two structureless methods *LazyLoc* and *E5+1* fall behind Hloc by approximately 10 percentage points on the smallest error thresholds, while being approximately 5 percentage points behind for the two larger error thresholds.

### Influence of mapping density

**Fig. 12:**
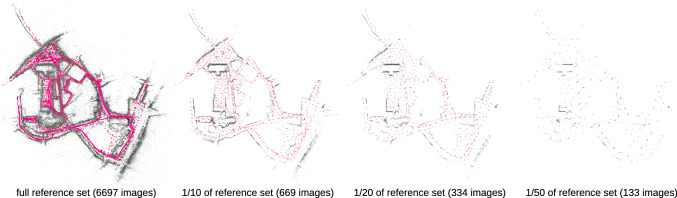
Comparison of the Aachen Day-Night v1.1 dataset (Sattler et al., 2018, 2012; Zhang et al., 2020) subsets used for the ablation in Table 10.

**Table 9:** Runtime analysis of scene representation updates. We are using global feature extraction and image retrieval to find corresponding reference images before building the structure-based scene representation. Note that the use of global features can be replaced by retrieving images based on their spatial distance or covisibility as we assume having known reference camera poses. The table contains two variants of structure-based methods: One extracts local features and discards the reference images in a preprocessing step and the other stores the original images and extracts the local features for matching between reference images on-the-fly.

method	switch local feature method	add image	remove image
$$\begin{array}{ll}\hbox {structure-based} \\ \hbox {(store images)}\end{array}$$	$$\begin{array}{ll}\text {N} \cdot \text {T}_\text {ext}\_\text {global} + \text {T}_\text {build}\_\text {ret} + \\ \text {N} \cdot \text {T}_\text {ext}\_\text {local} + \text {M} \cdot \text {T}_\text {match} + \\ \text {N} \cdot \text {T}_\text {build}\_\text {tracks} + \text {T}_\text {triang}\_\text {all}(\text {N})\end{array}$$	$$\begin{array}{ll}\text {T}_\text {add}\_\text {new}\_\text {image} + \text {T}_\text {ext}\_\text {global} + \\ \text {T}_\text {update}\_\text {ret} + (\text {K}+1) \cdot \text {T}_\text {ext}\_\text {local} + \\ \text {K} \cdot \text {T}_\text {match} + \text {K} \cdot \text {T}_\text {build}\_\text {tracks} + \\ (\text {I} + \text {J}) \cdot \text {T}_\text {triang}\_\text {single} + \text {T}_\text {update}\_\text {indices}\end{array}$$	$$\begin{array}{ll}\text {T}_\text {find}\_\text {image} + \text {T}_\text {remove}\_\text {image} + \\ \text {T}_\text {update}\_\text {ret} + \text {T}_\text {remove}\_\text {image}\_\text {observations} + \\ \text {I} \cdot \text {T}_\text {triang}\_\text {single} + \text {J} \cdot \text {T}_\text {remove}\_\text {point} + \\ \text {T}_\text {update}\_\text {indices}\end{array}$$
	$\sim$ hour(s)	$\sim$ minutes(s)	$\sim$ seconds(s)
$$\begin{array}{ll}\hbox {structure-based} \\ \hbox {(store features)}\end{array}$$	$$\begin{array}{ll}\hbox {not possible} \\ \hbox {(images not available)}\end{array}$$	$$\begin{array}{ll}\text {T}_\text {ext}\_\text {global} + \text {T}_\text {update}\_\text {ret} + \\ \text {K} \cdot \text {T}_\text {match} + \text {K} \cdot \text {T}_\text {build}\_\text {tracks} + \\ (\text {I} + \text {J}) \cdot \text {T}_\text {triang}\_\text {single} + \text {T}_\text {update}\_\text {indices}\end{array}$$	$$\begin{array}{ll}\text {T}_\text {update}\_\text {ret} + \text {T}_\text {find}\_\text {local}\_\text {features} + \\ \text {T}_\text {remove}\_\text {local}\_\text {features} + \\ \text {T}_\text {remove}\_\text {image}\_\text {observations} + \\ \text {I} \cdot \text {T}_\text {triang}\_\text {single} + \text {J} \cdot \text {T}_\text {remove}\_\text {point} + \\ \text {T}_\text {update}\_\text {indices}\end{array}$$
		$\sim$ minutes(s)	$\sim$ seconds(s)
$$\begin{array}{ll}\hbox {structureless} \\ \hbox {(store images)}\end{array}$$	$$\begin{array}{ll}\hbox { none} \\ \hbox {(extraction done at localization time)}\end{array}$$	$$\begin{array}{ll}\text {T}_\text {add}\_\text {new}\_\text {image} + \text {T}_\text {ext}\_\text {global} + \\ \text {T}_\text {update}\_\text {ret}\end{array}$$	$$\begin{array}{ll}\text {T}_\text {find}\_\text {image} + \text {T}_\text {remove}\_\text {image} + \\ \text {T}_\text {update}\_\text {ret}\end{array}$$
		$\sim$ seconds(s)	$\sim$ seconds(s)

Used notation:

$\bullet$ $\text {N}$ - number of reference images

$\bullet$ $\text {K}$ - number of retrieved reference images

$\bullet$ $\text {I}$ - number of 3D points to update (after adding / removing corresponding observations)

$\bullet$ $\text {J}$ - number of 3D points to add / remove

$\bullet$ $\text {T}_\text {ext}\_\text {global}$ - time needed to extract global feature from a single image

$\bullet$ $\text {T}_\text {build}\_\text {ret}$ - time needed to build the retrieval index from scratch

$\bullet$ $\text {T}_\text {ext}\_\text {local}$ - time needed to extract local features from a single image

$\bullet$ $\text {T}_\text {match}$ - time needed for local feature matching between a pair of images

$\bullet$ $\text {T}_\text {build}\_\text {tracks}$ - time needed to collect corresponding keypoints among images

$\bullet$ $\text {T}_\text {triang}\_\text {all}(\text {N})$ - time needed for triangulation from N reference images (using DLT)

$\bullet$ $\text {T}_\text {add}\_\text {new}\_\text {image}$ - time needed to add a single image (and its pose) into the scene representation

$\bullet$ $\text {T}_\text {update}\_\text {ret}$ - time needed to update the retrieval index without rebuilding

$\bullet$ $\text {T}_\text {triang}\_\text {single}$ - time needed for triangulation / retriangulation of a single point

$\bullet$ $\text {T}_\text {update}\_\text {indices}$ - time needed to update indexing between reconstruction elements

$\bullet$ $\text {T}_\text {find}\_\text {image}$ - time needed to find the reconstruction elements relevant to an image

$\bullet$ $\text {T}_\text {remove}\_\text {image}$ - time needed to remove a single image (and its pose) from the scene representation

$\bullet$ $\text {T}_\text {remove}\_\text {image}\_\text {observations}$ - time needed to remove the observations corresponding to an image from the reconstruction

$\bullet$ $\text {T}_\text {remove}\_\text {point}$ - time needed for removal of points with track length $< 2$

**Table 10:** Comparison of the best structureless methods with state-of-the-art structure-based method Hloc (Sarlin et al., 2019, 2020) on subsets of the Aachen Day-Night v1.1 (Sattler et al., 2018, 2012; Zhang et al., 2020). 1/*N* marks the fraction of reference images used for the evaluation. We use the top-10 images retrieved using the EigenPlaces (Berton et al., 2023) image-level descriptor. We report localization recalls (higher is better) at the pose thresholds of (0.25m, $2^{\circ }$) / (0.5m, $5^{\circ }$) / (5m, $10^{\circ }$).

			day			
method	matching	top-k	1/1	1/10	1/20	1/50
Hloc (Sarlin et al., 2019, 2020)	SP+LG	10	**87.0** / **94.8** / **98.5**	**68.9** / **82.4** / **90.2**	28.8 / 40.9 / 57.6	6.6 / 12.5 / 21.5
E5+1	SP+LG	10	76.6 / 88.3 / 97.5	53.9 / 70.5 / 88.8	33.4 / 47.5 / **73.9**	**12.7** / **22.0** / **38.1**
Local triang. - all	SP+LG	10	83.5 / 91.4 / 97.8	66.9 / 80.0 / 89.8	**41.0** / **54.4** / 71.4	12.5 / 21.0 / 36.9

			night			
			1/1	1/10	1/20	1/50
Hloc (Sarlin et al., 2019, 2020)	SP+LG	10	**70.2** / **87.4** / **97.4**	**46.6** / **70.7** / **88.5**	14.7 / 31.4 / 51.3	2.6 / 6.8 / 20.9
E5+1	SP+LG	10	61.3 / 85.9 / 96.9	34.0 / 58.6 / 83.8	12.0 / 28.3 / 61.8	3.1 / 9.9 / 22.0
Local triang. - all	SP+LG	10	66.5 / 84.3 / 96.3	45.0 / 70.2 / 85.9	**21.5** / **35.6** / **62.8**	**4.2** / **12.6** / **26.2**

Best-performing methods are given in bold

**Update complexity.** Table 9 shows a comparison of theoretical runtimes for structureless approaches with two options for structure-based methods. Both structure-based methods represent the scene through a 3D point cloud. In one case, the scene representation contains the original images and the corresponding approach extracts feature for the original images on-the-fly (for both scene updates and localization). In the second case, the scene representation stores pre-extracted local features and discards the original images. Both structureless and structure-based approaches have a set of update steps which need to be done in order switch to a different type of local feature, or to integrate new or remove old data and make the scene representation ready for localization. In the case of structureless methods, the update consists simply of adding or removing the posed image and updating the retrieval index. The update overhead of structure-based approaches is undeniably higher due to the set of steps, that might be, in summary, called retriangulation, which in practice consists of multiple steps, as shown in Table 9. As such, structureless methods require significantly less time to build / update the scene representation, *i.e.*, less time is required before the representation can be used for localization. Yet, the ease and speed of structureless methods updates comes at the price of a longer localization time. Therefore, each of the two approaches has its own possible use cases. Structure-based methods might be more suitable for static scenes with requirements for highly accurate localization, while structureless methods might be convenient in use cases which need fast deployment or frequent updates to the scene representation. An example of the latter case is construction sites, where the scene can change frequently (large structures being removed or built, and machines being moved). The new state of the scene can be captured by a surveillance camera, an AR headset of a worker, or a construction robot, and immediately used for map update.

Overall, we can see that structureless approaches can be competitive compared to structure-based methods. Coupled with the flexibility of their scene representation, structureless approaches thus represent an interesting alternative to the currently predominant structure-based methods.

To evaluate the influence of reference image sampling density, we followed the experiments presented in Panek et al. (2025) and generated multiple subsets of the Aachen Day-Night v1.1 dataset (Sattler et al., 2018, 2012; Zhang et al., 2020) reference image set. As we wanted a uniform coverage of the scene with the reference views, we used a greedy algorithm, which iteratively picks the camera farthest from all the cameras selected so far, until the required number of views is sampled. The algorithm also takes into account the relative angles between the cameras by adding a constant distance bonus (in our case $b_\text {max} = 100 m$) to the distance score if the relative angle is $\ge 90^\circ$. If the relative angle is in the range of $\langle 0^\circ , 90^\circ )$, we calculate the bonus as $(\alpha / 90^\circ )^2 \cdot b_\text {max}$. The resulting subset was used for both structureless localization and for point cloud triangulation for structure-based methods. We show the comparison of different dataset subsets in Fig. 12.

The results of the experiment, presented in Table 10, show that all methods suffer from sparsification of the reference image set, however, the accuracy of the structure-based Hloc (Sarlin et al., 2019, 2020) method decreases faster with the level of downsampling than the accuracy of the structureless methods. Comparison between structure-based and structureless localization pipelines on different datasets can be found in Panek et al. (2025).

## Lessons Learned

This section summarizes the main observations from the experiments, giving a quick guide to different design choices.

- The two best-performing methods are *Local triang. - all* (better on outdoor datasets) and *E5+1* (slightly better indoors) (see Tabs. 2, 3, 4 and 5).
- In average, the two best-performing local feature matchers are RoMa (better outdoors) and MASt3R (better indoors) (see Figs. 4, 5, 6, 7, 8, 9 and  10).
- The 3Pt+depth relative pose solver gives better results for pose triangulation (*Ess. mat.*) than the 5Pt solver. *LazyLoc* is not clearly better or worse than the two *Ess. mat.* approaches (see Tabs. 2, 3, 4 and 5).
- The opposite can be seen in the case of the semi-generalized approaches, where *E5+1* outperforms *E3+1* (see Tabs. 2, 3, 4 and 5).
- The choice of depth map for the 3Pt+depth and E3+1 pose solvers is not critical. Both perform similarly when used with Metric3D depth maps and MASt3R depth maps (see Figs. 5 and 8).
- When performing on-the-fly reconstruction, triangulating from all retrieved reference images (*Local triang. - all*) gives better and significantly more stable results than triangulating only from pairs of images (*Local triang. - pairs*) (see Tabs. 2, 3, 4 and 5).
- The tested relative pose regression methods do not yet achieve the accuracy of standard geometry-based approaches on large datasets (see Tabs. 1 and 2).
- Pose triangulation based on relative poses inferred by MASt3R (*Ess. mat. (MASt3R poses)*) gives the best results among the tested relative pose regression methods (see Table 1).
- Structure-based methods perform better than structureless methods in densely covered scenes, but degrade faster with reference image set sparsification (see Tables. 8 and 10).

## Conclusion

In this paper, we have provided a comprehensive overview and a detailed comparison of structureless visual localization approaches. Through extensive experiments, we have compared different families of structureless approaches. Our results show that more extensive geometric reasoning typically leads to better performance, with the best results being obtained by building local SfM models on the fly. However, the best accuracy-run-time tradeoff is provided by methods based on semi-generalized relative pose estimation. Our experiments did not reveal a single best localization or matching method, but they can serve as a reference for the reader to select the method based on the use case. We also looked into regression-based methods, which, from our evaluation, still need to mature to achieve the accuracy of more classical approaches. Compared to structure-based approaches, structureless methods are in general less accurate, but can perform comparably.

## Data Availability

This paper does not introduce any new data. The availability of the datasets used for the evaluation is as follows: $\bullet$ Aachen Day-Night v1.1 (Sattler et al., 2018, 2012; Zhang et al., 2020) - openly available at https://data.ciirc.cvut.cz/public/projects/2020VisualLocalization/Aachen-Day-Night/ $\bullet$ Extended CMU Seasons (Badino et al., 2011; Sattler et al., 2018) - openly available at: https://data.ciirc.cvut.cz/public/projects/2020VisualLocalization/Extended-CMU-Seasons/ $\bullet$ NAVER LABS Large-scale Localization Datasets in Crowded Indoor Spaces (Lee et al., 2021): - available upon request at https://www.naverlabs.com/en/datasets/requestDataset

## Footnotes

1 We use the distinction between structure-based and structureless (or "structure-less") methods employed in previous works (Bhayani et al., 2021; Dong et al., 2023, 2024; Humenberger et al., 2022; Lee et al., 2013; Panek et al., 2025; Pion et al., 2020; Zheng & Wu, 2015), which classify localization approaches based on whether they use some 3D model of the scene or not. This classification reflects the historical split in the literature between localization methods that used 3D models and place recognition methods that operated on the image level and mainly relied on image retrieval.

2 It might even not be necessary to delete images at all as outdated images will eventually not be retrieved or will not provide information when computing the pose of the query. A comparison of the steps necessary for the update of scene representation is presented in Table 9.

3 We do not match features between database images themselves.

4 Note that the initial alignment stage is needed to recover the scale of the local reconstruction, which is necessary for computing the additional points.

5 We used the AC-14 mesh model of the Aachen Day-Night v1.1 scene (Sattler et al., 2018, 2012; Zhang et al., 2020) provided by MeshLoc (Panek et al., 2022). The mesh is metric, in the sense that distances can be measured in meters.

6 Note that for the *Ess. mat.* approach, the scale of the depth maps is not important as we use relative poses up to the scale of the translation.
